# Multiparametric rapid screening of neuronal process pathology for drug target identification in HSP patient-specific neurons

**DOI:** 10.1038/s41598-019-45246-4

**Published:** 2019-07-03

**Authors:** Kristina Rehbach, Jaideep Kesavan, Stefan Hauser, Swetlana Ritzenhofen, Johannes Jungverdorben, Rebecca Schüle, Ludger Schöls, Michael Peitz, Oliver Brüstle

**Affiliations:** 1 Institute of Reconstructive Neurobiology, University of Bonn School of Medicine & University Hospital Bonn, 53127 Bonn, Germany; 2 LIFE & BRAIN GmbH, Cellomics Unit, 53127 Bonn, Germany; 3 German Centre for Neurodegenerative Diseases (DZNE), 72076 Tübingen, Germany; 4 German Centre for Neurodegenerative Diseases (DZNE), 53175 Bonn, Germany; 5 Memorial Sloan Kettering Cancer Centre, 10065 New York, United States; 6 Department of Neurodegenerative Diseases, University of Tübingen, 72076 Tübingen, Germany; 7 Present Address: Icahn School of Medicine, Mount Sinai, 10029 New York, United States

**Keywords:** Cellular imaging, Phenotypic screening, Neurodegeneration

## Abstract

Axonal degeneration is a key pathology of neurodegenerative diseases, including hereditary spastic paraplegia (HSP), a disorder characterized by spasticity in the lower limbs. Treatments for HSP and other neurodegenerative diseases are mainly symptomatic. While iPSC-derived neurons are valuable for drug discovery and target identification, these applications require robust differentiation paradigms and rapid phenotypic read-outs ranging between hours and a few days. Using spastic paraplegia type 4 (SPG4, the most frequent HSP subtype) as an exemplar, we here present three rapid phenotypic assays for uncovering neuronal process pathologies in iPSC-derived glutamatergic cortical neurons. Specifically, these assays detected a 51% reduction in neurite outgrowth and a 60% increase in growth cone area already 24 hours after plating; axonal swellings, a hallmark of HSP pathology, was discernible after only 5 days. Remarkably, the identified phenotypes were neuron subtype-specific and not detectable in SPG4-derived GABAergic forebrain neurons. We transferred all three phenotypic assays to a 96-well setup, applied small molecules and found that a liver X receptor (LXR) agonist rescued all three phenotypes in HSP neurons, providing a potential drug target for HSP treatment. We expect this multiparametric and rapid phenotyping approach to accelerate development of therapeutic compounds for HSP and other neurodegenerative diseases.

## Introduction

Axonal degeneration and neurite pathologies are key pathological hallmarks in a number of neurodegenerative diseases including spinal muscular atrophy^1,2^, amyotrophic lateral sclerosis (ALS)^3^, Charcot-Marie-Tooth disease^4^, multiple sclerosis^5^, Alzheimer’s disease^6^, Parkinson’s disease^7^, and HSP. HSP represents a heterogeneous group of disorders displaying progressive retrograde axonal degeneration of corticospinal motoneurons, manifesting by spastic paresis of the lower extremities^8–10^. The number of known mutations associated with HSP is steadily increasing. Until now, 78 different spastic gait disease-loci and 60 spastic paraplegia genes (SPGs) have been identified^10–13^. As indicated by the name, HSP is an inherited disease and has an overall prevalence of 1.8-9/10^5^, depending on the population studied^14,15^. Spastic paraplegia type 4 (SPG4) is the most frequent autosomal dominant subtype, being causative for >50% of the autosomal dominant cases and over 25% of all HSP cases^16^. Patients affected by SPG4 carry mutations in the *SPAST* gene encoding the microtubule-severing enzyme spastin^17^. There are two major isoforms of spastin, the 616 amino acid long M1 isoform (68 kDa) and the 530 amino acid long M87 isoform (60 kDa). While the M87 isoform is, in general, more abundantly expressed, the M1 isoform is thought to be predominantly expressed in the spinal cord^18^. SPG4 can be caused by a variety of mutations including missense, nonsense, splice site, deletions and insertions, mostly leading to a loss of protein expression, which indicates that the most common mode of action is haploinsufficiency^19^. A few cases where mutations affect the AAA ATPase domain of spastin have been associated with a dominant negative mode of action^18^. The key histopathological alteration in SPG4 patients are axonal swellings in the spinal cord, which represent a hallmark of HSP pathology^20^. This axonal swelling phenotype has been recapitulated in two mouse models expressing mutated spastin, either containing a premature stop codon or a splice site mutation, both resulting in a loss of protein^20,21^. While cultured cortical neurons derived from spastin mutant mice exhibit normal viability and neurite density^21^, spastin knockdown in developing zebrafish embryos leads to dramatic defects in axonal outgrowth of developing spinal motor neurons^22,23^. Riano *et al*.^24^ reported that spastin depletion in cultured mouse hippocampal neurons results in abnormal neuronal morphology, dystrophic neurites, axonal growth defects and reduced microtubule assembly rate. However, the disease mechanisms underlying the pathological changes found in SPG4 patients remain poorly understood, and no curative treatment has been identified so far. Apart from severing microtubules, spastin has been implicated in a number of other cellular processes including cytokinesis^25,26^, inhibition of BMP signalling^27^ and endosome trafficking^28^. In addition, the M1 isoform of spastin is located at the endoplasmic reticulum membrane and interacts with atlastin and REEP1, two other HSP-associated proteins, to take part in shaping the ER curvature^29^.

Insight into the pathogenesis of SPG4 and studies towards potential therapies would be greatly facilitated by experimental access to patient-specific human cells – an approach, which has become feasible with the advent of cell reprogramming and the generation of iPSCs. In this context, *in vitro* disease models featuring SPG4-specific pathophenotypes with short read-out times could vastly promote research towards desperately needed therapies to treat SPG4. Here we employ a protocol enabling the standardized production of cortical neurons from iPSCs to establish assay systems reporting key aspects of neurite pathology within very short read-out time periods. Pronounced changes in neurite length and growth cone morphology, as well as axonal swellings were detectable within only 1–5 days after cell plating, enabling screening applications for identification of therapeutic compounds for the treatment of SPG4.

## Results

### Standardized generation of cryopreservable iPSC-derived cortical precursors and differentiation into deep layer projection neurons

Fibroblasts from three members of one family carrying a heterozygous *SPAST* nonsense mutation and three unrelated, healthy controls were reprogrammed to a pluripotent state employing retroviruses or non-integrating Sendai viruses encoding *OCT4*, *KLF4*, *SOX2* and *C-MYC* to generate two iPSC lines per donor (Supplementary Table 1). Generated iPSC lines were extensively validated regarding pluripotency marker expression, genomic integrity via SNP analysis, the conformation of the disease-causing mutation, the absence of virus expression and the pluripotent differentiation potential via a teratoma assay and EB differentiation or Score card assay (Supplementary Table 1).

Considering that SPG4 mostly affects corticospinal neurons residing in layer V of the cortex, iPSCs were differentiated into deep-layer cortical neurons by modifying a previously published protocol^30^. This *in vitro* differentiation protocol recapitulates the sequential generation of deep and upper layer neurons as it is observed during physiological cortex development (VI to II).

**Figure 1:**
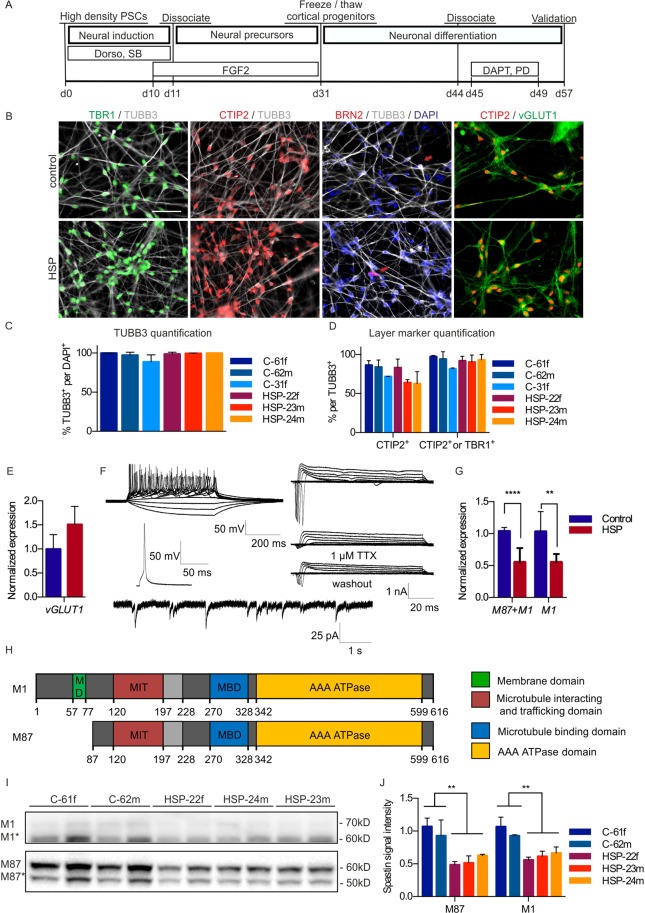
Differentiation of iPSCs into cortical progenitors and cortical neurons. (**A**) Timeline of cortical differentiation. (**B**) Immunofluorescence staining against the cortical layer markers TBR1 and CTIP2 as well as the vesicular glutamate transporter 1 (vGLUT1) confirms identity of cortical neurons. Scale: 50 µm. (**C**) Immunofluorescence stainings of neuronal marker TUBB3 and (**D**) layer markers TBR1 and CTIP2 were quantified on day 57 (C-61f: n = 4, C-62m: n = 3, C-31f: n = 3, HSP-22f: n = 6, HSP-23m: n = 4, HSP-24m: n = 3, C61f: 97.3%, C62m: 94.1%, C31f: 81.5%, HSP22f: 92.0%, HSP23m: 93.1%, HSP24m: 90.3%). Error bars show SD. (**E**) Q-PCR analysis confirms *vGLUT1* expression (control: n = 6, HSP: n = 6). (**F**) Top: Exemplary traces of multiple APs generated by 500 ms step-current injection (n = 4). Below: Single AP generated in response to 20 ms step-current injection. Right: Depolarising voltage steps elicited voltage-dependent TTX-sensitive fast inactivating inward and sustained outward currents (n = 7). Bottom: Representative trace of sPSC (n = 6). (**G**) Q-PCR of the M87 + M1 and M1 isoform of *SPAST* normalized to 18S levels and control (control: n = 6, HSP: n = 6). 2way Anova, **p < 0.01, ***p < 0.001. Error bars show SD. (**H**) Spastin is expressed as two major isoforms resulting from alternative translational start sites (M1 and M87) and two splice variants, in which exon 4 is skipped (M1* and M87*; exon 4 is shaded in light grey). (**I**) Spastin expression in control and SPG4 neurons. Western blot with spastin isoforms M87 and M87* after a short exposure time and M1 and M1* isoforms after prolonged exposure. To be able to show both isoforms, the M1 image had to be cut at the height of 60 kDa, to separate the M1 bands from the overexpressed M87 bands (for original Western blot data see Supplementary Fig. 6). (**J**) Quantification of isoforms M1 and M87 normalized to Ponceau total protein staining and control (C-61f: n = 4, C-62m: n = 4, C-31f: n = 4, HSP-22f: n = 4, HSP-23m: n = 4, HSP-24m: n = 4). Unpaired t-test, **p < 0.01. Error bars show SD. Figure adapted from corresponding PhD thesis^72^.

After 6 weeks of differentiation, the cortical cultures started to produce CTIP2-positive deep layer projection neurons. To ensure a homogeneous culture of neuronal layer V and VI neurons and to avoid generation of more superficial cortical layers and astrocytes by extended proliferation, the differentiation of the cortical progenitors into neurons was actively promoted by dissociation and subsequent treatment with PD0325901 and DAPT, which are known inhibitors of the MEK/ERK and Notch pathways, respectively (Fig. 1A).

Eight weeks after initiation of neural induction, the cultures were highly homogenous, consisting to 97.7 ± 4.8% of neurons, of which 92.1 ± 7.0% were projection neurons, positive for either the cortical layer V marker CTIP2 or the cortical layer VI marker TBR1 (Fig. 1C,D). In contrast, layer IV marker BRN2 was hardly detectable. Furthermore, the majority of neurons were positive for vGLUT1 on ICC and q-PCR level, confirming their glutamatergic identity (Fig. 1B,E). Interestingly, cortical cultures maintained for up to 52 days could still be cryopreserved as post-mitotic neurons, displaying post-thaw viability of 80% (data not shown). After three months of maturation on mitotically inactivated primary mouse astrocytes, both control and HSP neurons were able to fire repetitive action potentials in response to 500 ms depolarising current injection. Neurons exhibited fast inactivating inward current and sustained outward current in response to depolarising voltage steps. The fast inactivating inward current was reversibly blocked by the application of Tetrodotoxin. In addition, neurons showed spontaneous postsynaptic currents (Fig. 1F).

### SPG4 neurons exhibit spastin haploinsufficiency

The *SPAST* gene contains two translational start sites, which yield two main isoforms: M87 and the longer M1 isoform, which has an additional membrane binding domain close to the N-terminus. Splice variants lacking exon 4 exist for both isoforms, termed M87* and M1* (Fig. 1H). Q-PCR analysis of M1 and M87+M1 transcripts revealed a decreased expression of both, the M1 and M87 isoforms in SPG4 neurons (Fig. 1G). This finding was confirmed in Western blot analyses. As reported for primary CNS tissue^31^, the M87 isoform (60 kDa) and its splice variant were much stronger expressed than the M1 isoform (68 kDa) and its splice variant. In fact, the latter were only detectable after prolonged exposure (Fig. 1I). A quantification of spastin M87 and M1 bands, normalized to whole protein Ponceau staining, revealed a drastic reduction of spastin levels in SPG4 neurons to about 50% (Fig. 1J). The reduced expression of the *SPAST* transcript and the spastin protein together with the lack of detectable truncated protein specimens point to a spastin haploinsufficiency in our cortical neuronal population.

### 24-hour neurite outgrowth and growth cone assays reveal pronounced pathophenotypes in cortical SPG4 neurons

**Figure 2:**
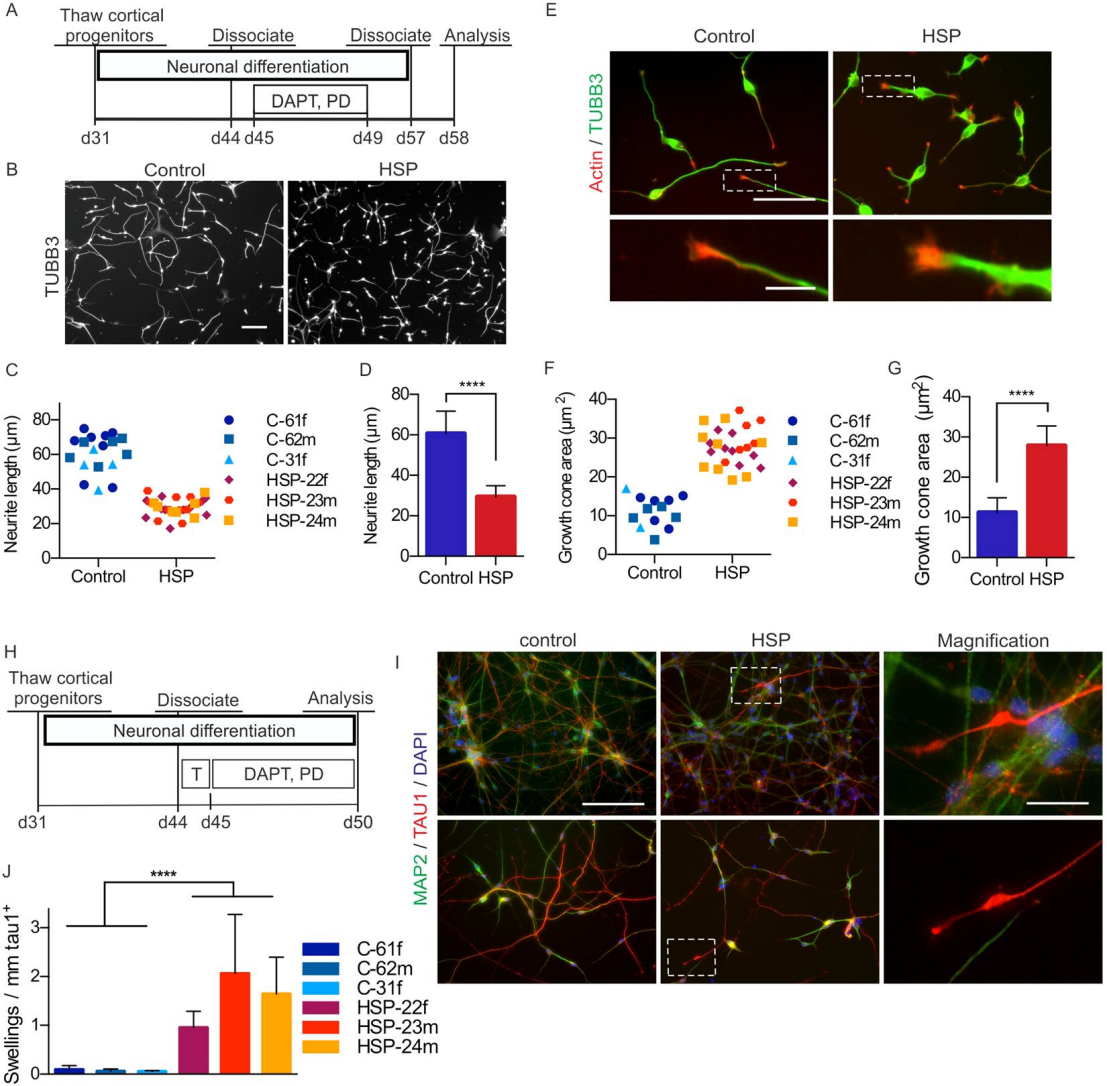
Short-term read-out of neurite outgrowth, growth cone formation and axonal swellings in iPSC-derived cortical neurons. (**A**) Timeline of the neurite outgrowth and growth cone assay. Cortical cultures were triturated to single cells and replated at low density on day 57. (**B**) Cells were fixed and stained for TUBB3 after 24 hours on day 58. Scale bar: 100 µm. (**C,D**) Quantification of the neurite outgrowth after 24 hours. SPG4 neurons exhibit a significant reduction in neurite length after 24 hours compared to controls. Data points depicted in (**C**) represent mean values of n = 18 (control) and n = 28 (HSP) biological replicates comprising ≥100 cells each. (**D**) Cumulative bar graph based on C; Mann-Whitney-U test; ****p < 0.0001; error bars show SD. (**E**) Immunocytochemistry for TUBB3 and actin on day 58. Scale bar: 50 µm. Insets show magnifications of typical growth cones. Scale bar: 10 µm. (**F,G**) SPG4 neurons exhibit a significant increase in growth cone area after 24 hours. Data points depicted in (**F**) represent mean values of n = 15 (control) and n = 25 (HSP) biological replicates comprising ≥100 cells each. (**G**) Cumulative bar graph based on (**F**); Mann-Whitney-U test; ****p < 0.0001; error bars show SD. (**H**) Timeline of the axonal swelling assay: neurons were stimulated with 3 nM taxol on day 44 and cultivated until day 50. (**I**) Cortical neurons were fixed and stained for the dendrite marker MAP2 and the axon marker TAU1 on day 50. SPG4 neurons exhibit prominent TAU1 positive axonal swellings. Scale bar: 100 µm. (**J**) Quantification of TAU1 positive axonal swellings. The number of TAU1 positive axonal swellings in patient neurons was significantly higher (mean of 1.537/mm) compared to that of control neurons (mean of 0.073/mm). Data are based on ≥100 cells per biological replicate (C-61f: n = 7, C-62m: n = 7, C-31f: n = 4, HSP-22f: n = 10, HSP-23m: n = 7, HSP-24m: n = 9); unpaired t-test; ****p < 0.0001; error bars: SD. Figure adapted from corresponding PhD thesis^72^.

Rapid phenotypic read-outs on neurons should ideally be combined with passaging and replating as well as cryopreservation of fully differentiated cells, followed by a short follow-up time during which the phenotype is robustly detectable. We explored this concept by dissociating and replating neuronal cortical cultures at day 57 of neuronal differentiation for phenotypic assessment 24 hours later. We focussed on neurite length and growth cone size, two parameters expected to be affected by altered microtubule severing activity^32^ (Fig. 2A). Indeed, SPG4 neurons displayed a prominently visible reduction in neurite length (Fig. 2B). Total length of neurites per neuron ranged from 39 µm to 75 µm in control neurons and from 17 µm to 39 µm in patient neurons with slight variations between individual donors and experiments (Fig. 2C). Interestingly, mean neurite lengths of patient neurons clustered very closely together, whereas neurons from healthy control donors showed more variability (Fig. 2C). Overall, neurite length in patient neurons was drastically decreased by 51% (control: 60.6 ± 11.1 µm; SPG4: 29.4 ± 5.4 µm; Fig. 2D).

Since growth cones mainly consist of actin filaments closely interacting with microtubules, which stabilize and shape growth cones^33,34^, we went on to investigate differences in growth cones. Upon staining of actin filaments, morphological differences between growth cones of patient and control neurons were identified. In particular, growth cones of patient neurons were increased in size compared to growth cones of control neurons (Fig. 2E). To assess differences in growth cone size, the area taken up by actin filaments, located at the end of neurites, was quantified (for details see Methods). Quantification revealed that the growth cone area after 24 hours of outgrowth ranged from 4 µm^2^ to 17 µm^2^ in control neurons and from 20 µm^2^ to 37 µm^2^ in patient neurons, varying in independent experiments (Fig. 2F). The overall mean growth cone area of controls was 11.2 ± 0.9 µm^2^, that of SPG4 neurons 27.9 ± 1.0 µm^2^ (Fig. 2G). In conclusion, spastin haploinsufficiency leads to drastically enlarged growth cones.

### Capturing SPG4-associated axonal degeneration in a 5-day-assay

Retrograde degeneration of the corticospinal neurons with axonal swellings in the post-mortem spinal cord is the histopathological hallmark of SPG4, which can therefore be classified as axonopathy. Since we concentrated our analysis on fast assays, we used the fact that treatment of the cultures with low doses of taxol, a microtubule stabilizer, expedites axon formation^35,36^. Without taxol treatment axons showed TAU1-positive staining only after prolonged maturation until day 75, yielding very dense, hardly quantifiable networks (Supplementary Fig. 2). However, by treating neuronal cultures with low doses of taxol on day 44, a complex but thin neuronal network containing MAP2 positive dendrites and TAU1 positive axons formed by day 50. These cultures were used as basis to investigate an axonal phenotype in the SPG4 cortical neurons (Fig. 2H). Indeed, axonal swellings were identified in TAU1 positive patient axons but not in MAP2 positive dendrites and only rarely in axons of control neurons. The measured normal axon width was always below 1 µm in diameter, while observed swellings varied in diameter from 1 to 7 µm and were mostly located at the very distal end of the axon (Fig. 2I, indicated boxes). Axons from control cultures exhibited only 0.07 ± 0.06 swellings per mm axon, whereas axons from SPG4 cultures showed a pronounced increase of swellings which amounted to 1.54 ± 0.99 swellings per mm (Fig. 2J).

### Neuronal subtype specificity of fast SPG4 phenotypes

**Figure 3:**
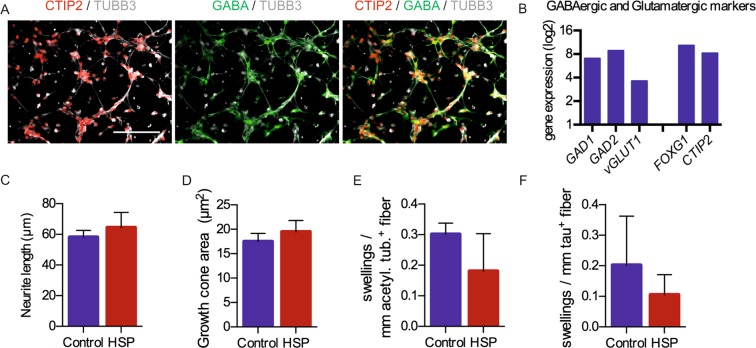
Short-term read-out of neurite outgrowth, growth cone formation and axonal swellings in iPSC-derived GABAergic neurons. (**A**) Immunofluorescence staining for the neurotransmitter GABA and the transcription factor CTIP2, expressed in TUBB3 positive neurons. (**B**) Affymetrix expression data (log2) of two control GABAergic differentiations on day 37 (the expression data is typically interpreted as follows: 0–4:  sub-threshold expression levels, 4–8: medium expression, >8: highly expressed), confirms the expression of the GABA producing enzymes *GAD1* and *GAD2*. The GABAergic cultures express the forebrain marker *FOXG1* and the transcription factor *CTIP2*, which is both present in the cortex and the striatum. (**C**) Quantification of the mean neurite outgrowth after 24 hours. (**D**) Quantification of the mean growth cone area after 24 hours. Data shown in (**C**,**D**) are based on n = 8 (control) and n = 10 (HSP) biological replicates (≥200 cells per replicate). (**E**) Quantification of acetylated tubulin-positive swellings. (**F**) Quantification of tau-positive swellings. Data shown in (**E**) and (**F**) are based on n = 4 (control) and n = 6 (HSP) biological replicates (≥100 cells per replicate). All quantifications in this figure were performed using the GE InCell Developer toolbox. Figure adapted from corresponding PhD thesis^72^.

To test whether these observed phenotypes were specific to glutamatergic cortical neurons, GABAergic forebrain neurons were generated as an alternative population. These inhibitory SPG4 neurons were generated by a similar protocol, which was adapted by increasing dual SMAD inhibitor concentrations and shortening the proliferation phase (Supplementary Fig. 3A) and yielded post-mitotic neurons expressing GABA and the transcription factor CTIP2 already after 37 days (Fig. 3A), markers typically observed in cortical interneurons^37^. The cellular identity was further studied using microarray-based expression data obtained from control neurons, which confirmed the expression of GABA producing enzymes *GAD1* and *GAD2* and low levels of *vGLUT1*. Among other expressed markers were the forebrain marker *FOXG1* and the transcription factors *CTIP2*, which can be present in the cortex in glutamatergic or GABAergic neurons as well as in the striatum^37,38^ (Fig. 3B). The generated GABAergic neurons expressed the GABAergic subtype marker somatostatin (*SST*) and several striatal markers but showed low expression of typical markers of glutamatergic cortical progenitors and neurons, *TBR1* and Eomesodermin/TBR2 (*EOMES*) (Supplementary Fig. 3). In conclusion, this alternative neuronal population consists of forebrain interneurons with an expression profile overlapping with the classical cortical glutamatergic neurons and is thus an excellent neuronal population for comparison. After three months maturation on mitotically inactivated primary mouse astrocytes, GABAergic neurons were electrophysiologically active (Supplementary Fig. 3C).

Due to a shortening of the proliferation phase, GABAergic neurons emerged around three weeks earlier than glutamatergic neurons, yielding a comparable age of GABAergic neurons on day 37 and glutamatergic neurons on day 57. Thus, we set out to investigate neurite outgrowth and growth cone assays of the forebrain GABAergic neurons on day 37 (Supplementary Fig. 4A). Both, control and SPG4 GABAergic forebrain neurons exhibited TUBB3 positive neurites and actin positive growth cones after 24 hours (Supplementary Fig. 4B). Interestingly, the neurite length and the growth cone area of SPG4 GABAergic neurons did not differ compared to control GABAergic neurons after 24 hours (Fig. 3C,D). Next, we set out to induce axonal structures as was done in the glutamatergic cultures using taxol treatment (Supplementary Fig. 4C). However, treatment with low doses of taxol as described in glutamatergic cultures, did not induce TAU1 positive axons in GABAergic neurons. Therefore, cultures were stained for total TAU and stabilized, acetylated tubulin, which has been used in previous studies to quantify swellings^39^ (Supplementary Fig. 4D). Interestingly, both control and SPG4 GABAergic neurons exhibited only a very low level of swellings, which did not differ between the groups (Fig. 3E,F).

In conclusion, the three early phenotypes observed in SPG4 glutamatergic neurons could not be observed in GABAergic neurons and are therefore subtype specific. We hypothesized that spastin levels could be differentially regulated in GABAergic forebrain neurons, leading to amelioration of the phenotypes compared to those observed in glutamatergic cortical neurons. To test this hypothesis, the cultures were used to analyse the expression of M1 and M87 *SPAST* transcripts. Quantification of *SPAST* mRNA levels confirmed the haploinsufficiency of both glutamatergic and GABAergic SPG4 cultures and revealed that GABAergic neurons of SPG4 patients had significantly higher M1 *SPAST* levels compared to glutamatergic neurons of SPG4 patients, even though the *SPAST* levels in GABAergic control neurons were similar to those of glutamatergic control neurons (Supplementary Fig. 5). Thus, the reduced spastin expression level in patient neurons seems to be crucial for the development of a disease phenotype *in vivo* and *in vitro*.

### Exploiting rapid evolvement of SPG4 pathophenotypes for drug testing

Taking advantage of the rapidly developing *in vitro* pathophenotypes in cortical excitatory neurons we set out to assess whether the developed assays lend themselves to drug testing. To achieve a higher throughput, the assays were transferred to a 96-well setup, which was combined with a semi-automated image acquisition and analysis pipeline (for details see Methods).

Based on preclinical and clinical reports we selected a small collection of compounds, which were implicated in modulating pathophenotypes associated with neurodegeneration. The histone deacetylase inhibitor scriptaid has neuroprotective properties and has been proven to be beneficial in models of ALS, Parkinson’s and Alzheimer’s disease^40,41^. The microtubule destabilising drug vinblastine has previously been used to successfully counteract axonal swellings both, in a mouse model of SPG4^42^ and in SPG4 patient iPSC-derived neurons^43^. TRO19622 acts as inhibitor of the mitochondrial permeability pore (mPTP) preventing a response to oxidative stress^44^. It has been proven to be neuroprotective in several disease models and tested in phase III clinical trials for the treatment of ALS^45,46^. Since we discovered a novel phenotype in patient neurons, i.e., the enlargement of growth cones, we included the actin destabilising drug latrunculin B in this compound testing^47,48^. Furthermore, latrunculin B has previously been reported to promote neurite elongation^49^. Since spastin has a known function as inhibitor of BMP signalling, we decided to also test the small molecule BMP inhibitor dorsomorphin and its highly selective, small molecule analogue DMH1^50,51^. In addition, altered lipid homeostasis and insufficient lipid droplet formation have been implicated in the pathogenesis of SPG4^52^. The LXR agonist GW3965 promotes the formation and expansion of lipid droplets and shows good blood-brain barrier penetration^53,54^. Furthermore, GW3965 has been shown to exhibit neuroprotective properties in ischemia models^55^.

**Figure 4:**
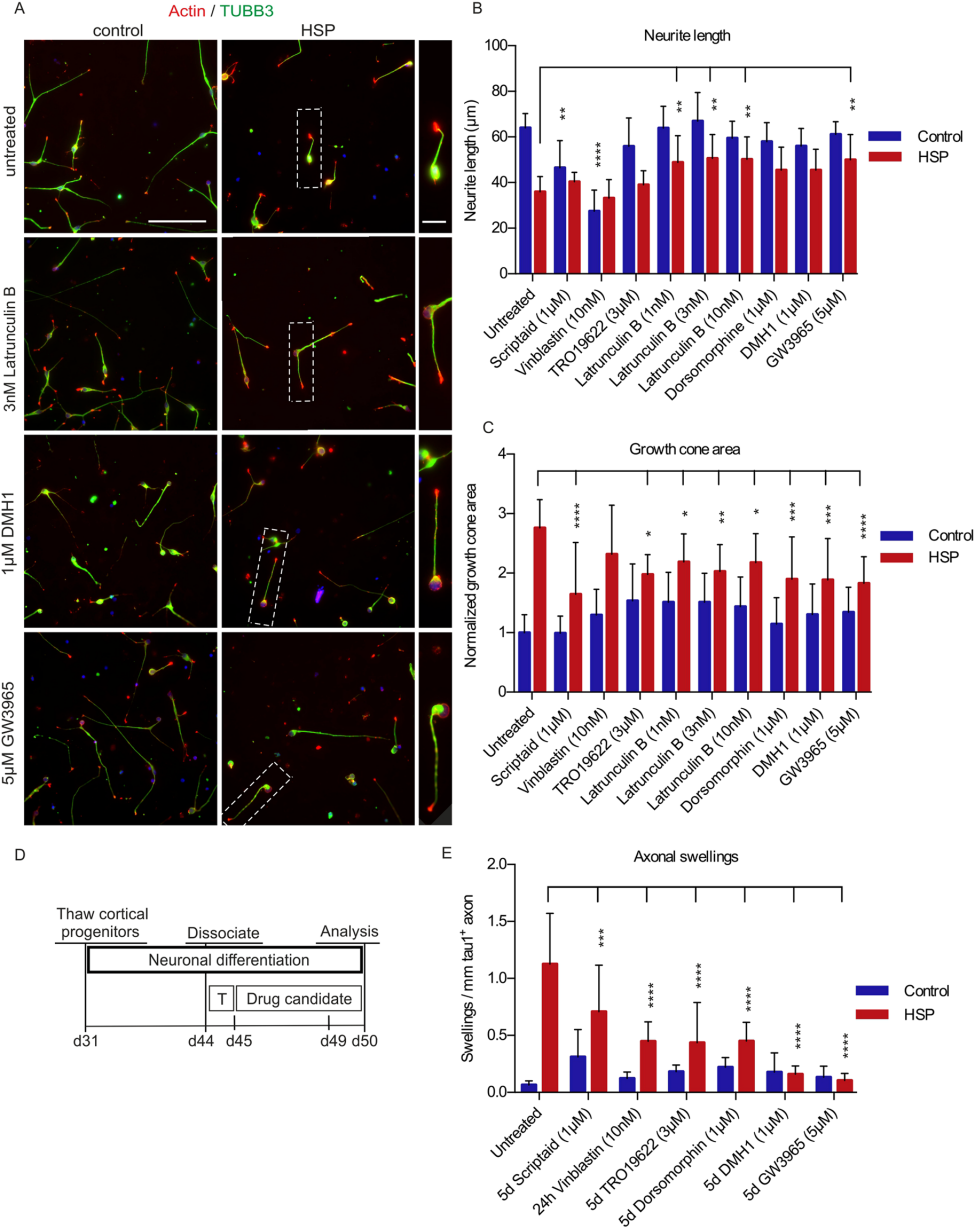
Proof-of-principle compound assessment using semi-automated image acquisition and analysis. (**A**) Cortical neurons were dissociated and singularized on d57 and seeded on 96-well plates pre-treated with small molecules. After 24 hours, plates were fixed and stained against TUBB3, actin and DAPI. (**B**) The mean neurite length of control (C-61f, C-62m, C-31f) and HSP (HSP-22f, HSP-23m, HSP-24m) neurons and the effects of selected drug treatments after 24 hours were quantified using the InCell Developer toolbox. (**C**) The mean growth cone area in control (C-61f, C-62m, C-31f) and SPG4 (HSP-22f, HSP-23m, HSP-24m) neurons and the effects of drug treatments after 24 hours were quantified using the CellProfiler software. Data shown in (**B**,**C**) are based on n = 10 (control) and n = 14 (HSP) biological replicates (≥100 cells per replicate). (**D**) Timeline for drug treatment in the axonal swelling assay. T stands for addition of 3 nM taxol overnight. All drugs were incubated for 5 days and added on day 45, except vinblastine which was incubated for 24 hours and added on day 49. (**E**) Quantification of axonal swellings in control (C-61f, C-62m, C-31f) and HSP (HSP-22f, HSP-23m, HSP-24m) neurons and the effects of drug treatments after five days or 24 hours, respectively, normalized to mm/tau^+^ axon. All tested drugs reduced axonal swellings significantly in SPG4 neurons. However, the best effect, combined with normal neuronal morphology was achieved by GW3965 and DMH1 treatment. Data shown in (**E**) are based on n = 5 (control) and n = 9 (HSP) biological replicates (≥100 cells per replicate). 2way ANOVA; ***p < 0.001, ****p < 0.0001; error bars show SD. Figure adapted from corresponding PhD thesis^72^.

In the neurite outgrowth assay, the actin destabilising drug latrunculin B and the LXR agonist GW3965 led to a significant increase in neurite length of SPG4 neurons, whereas DMH1, dorsomorphin and TRO19622 had no significant effect on the neurite length of either patient or control neurons. In contrast, vinblastine and scriptaid treatment led to decrease of neurite length in control neurons (Fig. 4A,B). In the growth cone assay, several of the tested drugs (scriptaid, TRO19622, latrunculin B, dorsomorphin, DMH1, GW3965) resulted in a significant reduction of growth cone areas of SPG4 neurons (Fig. 4A,C). Taken together, two drugs (Latrunculin B and GW3965) were found to induce an increase of neurite length and a reduction of growth cone area in SPG4 neurons, while maintaining a normal neuronal morphology in both patient and control neurons.

For the axonal swelling assay drug treatment was applied daily from day 45 to day 50 (Fig. 4D). All compounds were applied for 5 days, except vinblastine, which was only added for 24 hours because 5-day treatment resulted in neuronal demise. Interestingly, all tested drugs led to a significant reduction of axonal swellings in patient cultures compared to untreated and DMSO treated controls (Fig. 4E). This effect was most pronounced for DMH1 and GW3965, without a visible impact on overall neuronal morphology. In summary, of all the compounds tested the LXR agonist GW3965 was able to rescue all three pathophenotypes detected in SPG4 neurons and led to an increase of neurite outgrowth, a decrease of growth cone area and a reduction of axonal swellings. Furthermore, we detected no significant effects of GW3965 on control neurons.

## Discussion

**Figure 5:**
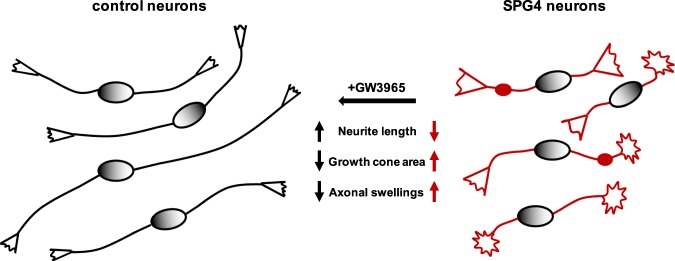
Synopsis of cellular pathophenotypes in human SPG4 neurons and their drug-mediated rescue.

Numerous neurodegenerative diseases are associated with axonal degeneration and neurite pathologies. While iPSC-based models have been successfully used to recapitulate these pathophenotypes, the read-out times required for their analysis typically range from few weeks up to several months. However, in order to remain feasible for the assessment of pharmaceutical compounds and screening applications, much shorter read-out times are required. Here, we developed an assay system, which detects a reduction of neurite length of SPG4 cortical neurons 24 hours after plating, and at the same time, an increase in growth cone size – a phenotype so far not reported for SPG4 neurons. Furthermore, axonal swellings, a pathological hallmark of SPG4^20^, could be recapitulated *in vitro* just five days after plating. In addition, a compound screen employing these fast read-outs identified the LXR agonist GW3965 as a candidate able to rescue all three phenotypes (Fig. 5).

In this study, a human iPSC-based SPG4 model was developed and very early phenotypes were identified. We report a drastic neurite outgrowth deficit in SPG4 neurons with a reduction of neurite length by 51% 24 hours after plating of two-week-old neurons. A previously published iPSC disease model reported a decrease in neurite length of 40% in human SPG4 neurons after 6 weeks of maturation without the possibility of replating^39^. Thus, the read-out time in our study is immensely accelerated. In addition, we identified enlargement of growth cones by 60% as a novel phenotype that can be measured simultaneously to the neurite outgrowth. Considering the role of spastin as microtubule severing enzyme, it is conceivable that the observed neurite outgrowth deficit is due to overly stabilized microtubule. We speculate that there is a connection between the neurite outgrowth deficit and the enlarged growth cones. Overly stabilized growth cones might inhibit microtubule protrusion leading to shorter neurites or *vice versa*, overly stabilized microtubules might not be able to push the growth cones forward, which, in consequence, grow larger^32^.

In a third phenotypic assay the formation of axonal swellings was accelerated by a low dose of taxol treatment, a microtubule stabilising drug which was reported to lead to a four-fold increase of the axonal marker TAU1 in mouse hippocampal neurons^36^. In our setting, a single overnight stimulation with a low dose of taxol led to axonal swellings in one-week old neurons five days after treatment. The axonal swelling phenotype itself has been previously shown in two human SPG4 iPSC models after six weeks of maturation^39,43^. In contrast to these conventional axonal swelling assays, our accelerated assay shows swellings six days after plating, which is compatible with high-throughput drug testing. The emergence of axonal swellings might be due to the deficient microtubule severing caused by spastin haploinsufficiency, which might lead to an abundance of disorganized microtubules. Disorganized microtubules within neurite swellings were previously demonstrated using electron microscopy^39^.

The disease phenotypes observed here *in vitro* developed much faster than one could have expected from the clinical phenotype of HSP patients, where the emergence of symptoms takes decades. The development of early phenotypes *in vitro* might be explained by the fact that the cells are cultured in a reduced environment lacking the endogenous niche, which is composed of different cell types such as astroglia that provide trophic support and nutrients to the neurons. This lack of support and the unphysiologically high atmospheric oxygen level might impose stress on the cultured neurons. Thus, phenotypes such as the neurite outgrowth deficit that might be compensated for by the surrounding tissue and therefore do not develop *in vivo* can emerge *in vitro*. However, it might also be possible that SPG4 patients already have a slight developmental defect or a reduced regeneration potential of corticospinal neurons compensated *in vivo* until the disease onset.

Spastin is ubiquitously expressed, but the patient phenotype is caused by degeneration of a very distinct set of neurons, i.e. cortical deep layer projection neurons. This points towards a neuronal subtype-specific disease susceptibility. To test whether this is also the case *in vitro* and whether the neuronal subtype is important for the observed phenotype, we generated inhibitory forebrain neurons. None of the phenotypes observed in glutamatergic cortical neurons could be recapitulated in GABAergic forebrain neurons, supporting the notion that the disease only acts on a specific subset of neurons. This might be explained by the observation that in contrast to glutamatergic neuronal cultures, GABAergic neuron exhibited significantly higher expression of the *SPAST* M1 isoform. This dosage phenomenon might explain the absence of a phenotype in GABAergic patient cultures and points towards a critical role of M1 spastin insufficiency in SPG4 pathology. This hypothesis is supported by data that overexpression of the M1 isoform has a stronger effect in reversing SPG4 phenotypes than overexpression of M87^39^. Even though the M87 isoform is ubiquitously expressed and has the most abundant expression, the enzyme functions as a hexamer, which can consist of various combinations of M87 and M1 isoforms^56^. However, only the membrane domain of the M1 isoform can localize the hexamer to the ER membrane. Furthermore, expression of the M1 isoform is elevated within the corticospinal pathway of rats and has thus been reported to be the more disease relevant isoform^31^. Since the M87 isoform is more abundantly expressed, the M1 isoform might be the limiting factor, which cannot be easily compensated for.

The protocol used for generating a standardized and highly purified population of cortical neurons is scalable, with the option to cryopreserve the emerging cortical neurons. Together with the short read-out times required for assessing the different pathophenotypes after replating the cells, these features provide an optimal basis for drug screening applications. For that reason, all three phenotypic assays were transferred to a semi-automated 96-well-setup to study, as an exemplar, a small collection of compounds potentially modulating HSP-associated disease pathways. Neurite length and the growth cone area were quantified automatically, whereas neurite swellings were counted manually and normalized to the automatically quantified axon length. Future studies would benefit from fully automated assessment of swellings, which might be possible by visualising swelling-specific accumulation of cargo such as mitochondria or lysosomes. From the biological side, interindividual variability could be reduced by using control samples derived from healthy relatives of the patients’ families or even isogenic controls generated by correction or introduction of the disease mutation via genome editing.

Among the various compounds assessed in the context of this proof-of-concept application, the LXR agonist GW3965 stood out as the only compound able to rescue all three pathophenotypes studied. GW3965 has been reported to modulate cholesterol metabolism and transport, lipogenesis and protection from cholesterol overload^57^. It acts selectivity on LXR-beta, the receptor subtype mostly expressed in the brain^58^. Activation of LXR leads to upregulation of pathways involved in cholesterol synthesis, and to an even higher extent to cholesterol efflux out of the cells^59^. Thus, LXR activation might be required to drain excess sterols and lipids from the central nervous system to prevent an overload. The identification of GW3965 as rescue molecule may point towards a central role of cholesterol metabolism in SPG4. Interestingly, in SPG5, a different subtype of HSP, oxysterols like 27-hydroxycholesterol (27-HC) accumulate and cause retrograde degeneration of corticospinal neurons^60,61^.

Several other lines of evidence suggest an important role for cholesterol homeostasis in corticospinal neuron degeneration. First of all, it has been reported that the spinal cord exhibits a higher cholesterol concentration compared to residual CNS. Secondly, mice with inactivated LXR-beta show lipid accumulation in the spinal cord and specific motoneuron degeneration, mimicking ALS^62^. Moreover, it has been reported that cortical neurons have less cholesterol than hippocampal neurons and only tolerate low levels of cholesterol fluctuation. Studies in mice revealed that both, increased and decreased levels of cholesterol result in decreased neurite outgrowth specifically in cortical neurons^63^. Furthermore, cholesterol concentrations are distinctly regulated in growth cones, which contain significantly less cholesterol compared to the overall plasma membranes of mature rat neurons^64^. Mechanistically, accumulation of cholesterol derivatives in SPG4 patients could damage the axons of the particularly sensitive corticospinal neurons, leading to retrograde degeneration. Several LXR agonists have entered phase I clinical studies and are of interest for numerous diseases (cardiovascular disease, cancer, Alzheimer’s disease, Parkinson’s disease, multiple sclerosis, Huntington’s disease). Furthermore, novel, even more specific LXR-beta agonists are being developed that could be suitable for clinical application in patients and maybe even SPG4 in the near future^65–68^.

In summary, our study shows that pronounced *in vitro* phenotypes can be observed in SPG4 neurons already after short read-out times compatible with a compound screening scenario. The fact that an LXR agonist can rescue these *in vitro* phenotypes might hint to a cholesterol-associated disease mechanism and thus warrant further evaluation of this substance class as potential therapeutics.

## Materials and Methods

### Reprogramming of patient and control fibroblasts

Skin fibroblasts of three SPG4 patients and three healthy unrelated controls were reprogrammed into iPSCs. All cell lines were infected with the classic Yamanaka reprogramming factors: *OCT4*, *SOX2*, *KLF4* and *C-MYC*. iPSCs from the female patient HSP-22f and the female control C-31f were generated using retroviruses, while all other cell lines (HSP-23m, HSP-24m, C-61f, C-62m) were infected with non-integrating Sendai RNA viruses to induce pluripotency. All cell lines were initially generated on mouse irradiated feeder-cells and later switched to feeder-free conditions. The patients participating in this study belong to one family and carry an identical nonsense mutation in the *SPAST* gene: c.577C > T p.Q193*. The study was approved by the Ethics Committee of the Medical Faculty of the University of Bonn (approval number 275/08), and informed written consent was obtained from the patients and healthy donors. All experiments were performed in accordance with German guidelines and regulations.

### Differentiation of induced pluripotent stem cells into glutamatergic cortical neurons

The cortical differentiation was adapted from a previously published protocol^30^. Specifically, iPSCs were cultured in mTeSR (StemCell Technologies) or StemMACS™ iPS-Brew (Miltenyi Biotec) and split with EDTA during maintenance culture^69^. Undifferentiated iPSCs were dissociated with accutase and seeded as single cells at a density of 1 × 10^6^ cells per cm^2^ in iPSC medium with 10 µM ROCK inhibitor (RI) Y-27632 (Tocris). The next day, the medium was switched to GLUT neural induction medium (1:1 DMEMF12/N2:Neurobasal/B27, 1 µM Dorsomorphin/200 nM LDN-193189, 10 µM SB431542). On day 10, the neural induction medium was supplemented with 20 ng/ml FGF2 to accelerate neural rosette growth. On day 11, the cultures were split by incubating accutase for 15 min. Obtained cell clumps were seeded on Matrigel (MG)-coated 6-well plates in N2/B27 medium (1:1 DMEMF12/N2:Neurobasal/B27) with 20 ng/ml FGF2 and 10 µM RI using a split ratio of 1:3. On days 12 and 13, the medium was replaced with N2/B27 medium. From day 14 onward, cells were cultured in N2/B27 medium supplemented with 10 ng/ml FGF2 and 100 ng/ml heparin. On day 17 and day 22, the cultures were dissociated with accutase and seeded 1:2 on MG-coated plates for further propagation. On day 31, cortical neural precursor cultures were frozen down as one batch in ice-cold freezing medium (90% KOSR, 10% DMSO). Cortical neural precursors were thawed for further maturation and seeded in N2/B27 medium supplemented with 10 µM RI on MG-coated plates (0.5 mio cells per cm^2^). On day 44, cultures were dissociated one more time and seeded for maturation. On the following day, the medium was replaced by N2/B27 medium with 10 µM PD0325901 and 10 µM DAPT to accelerate differentiation of persisting precursors. The medium was renewed on day 47. The cultures were mitotically inactivated with 5 µM AraC (Cytosine β-D-arabinofuranoside hydrochloride) on day 49. Up to the analysis cells were cultured further in N2/B27 medium with medium changes every other day, without aspirating the medium completely.

### Differentiation of induced pluripotent stem cells into GABAergic forebrain neurons

To generate an alternative neuronal population, GABAergic forebrain neurons were generated. To this end, iPSCs were cultured in E8 medium during maintenance^69^. IPSCs were dissociated with accutase and seeded at a density of 0.2 × 10^6^ per cm^2^ in E8 medium supplemented with 10 µM RI. The following day, the medium was replaced by neural induction medium (1:1 N2:B27, 500 nM LDN-193189, 15 µM SB431542). The neural induction medium was supplemented with 20 ng/ml FGF2 to accelerate neural rosette formation, on day 9. The next day, cultures were split using accutase for 10 min and seeded in N2/B27 medium with 20 ng/ml FGF2 and 10 µM RI, 1:3 onto MG-coated 6-well plates. 20 ng/ml FGF2 were kept in the medium until day 12. From day 13 onward, the cells were cultured in N2/B27 medium, which was changed every other day. Neural precursors were dissociated and frozen down as one batch on day 20. For maturation, frozen precursors were thawed and seeded in N2/B27 medium with 10 µM RI at a density of 0.5 × 10^6^ cells per cm^2^. On day 27, cultures already exhibited a large fraction of neurons and were seeded for final maturation on different MG-coated plates. On the next day, the medium was changed to neuro medium supplemented with 10 µM PD0325901 and 10 µM DAPT to force differentiation of remaining precursors. The same medium was refreshed on day 29. On day 31, the cultures were mitotically inactivated by treatment with 5 µM AraC, to prevent further proliferation. Up to the analysis, the cells were cultured further in N2/B27 medium with medium changes every other day, without aspirating the medium completely.

### Neurite outgrowth and growth cone assays

The neurite outgrowth and the growth cone assays were performed on cortical glutamatergic cultures on day 57 and GABAergic cultures on day 37, thus on post-mitotic neurons of 2 weeks of age. Neuronal cultures were dissociated to single cells using accutase supplemented with 10 µM RI for 60 minutes at 37 °C. After centrifugation, the cells were resuspended in N2/B27 medium supplemented with 10 µM RI and passed through a 40 µm cell strainer before seeding. To achieve low density-cultures with single neurons, 20.000 cells per cm^2^ were seeded on MG-coated 3.5 cm dishes or 96-well plates. Control and patient cell lines were split and plated simultaneously to avoid time differences. After exactly 24 hours, cultures were fixed with 4% PFA and stained with DAPI and ActinRed 555 (Thermo Scientific) and against TUBB3.

### Axonal swelling assay

Axonal swellings were analysed by immunocytochemical stainings against axonal TAU1 of 50-day old glutamatergic cortical cultures or 37-day old GABAergic cultures. Specifically, neural cultures were seeded in N2/B27 medium supplemented with 3 nM taxol and 10 µM ROCK inhibitor after dissociation on day 44 or day 27, respectively. The medium was removed the next day and replaced by N2/B27 medium supplemented with 10 µM PD0325901 and 10 µM DAPT, which was renewed on day 47 and day 49, or day 30 and day 32, respectively. For drug testing a similar timeline was implemented and drug treatment and solvent controls were applied daily from day 45 to day 50 (Fig. 4D). The only exception is a 24-hour treatment with vinblastine from day 49 to day 50, which was performed according to previously published results^43^. Cultures were fixed with 4% PFA and stained against TAU1 and MAP2 on day 50. GABAergic cultures were fixed on day 37 and were additionally stained against TAU, MAP2ab, Acetylated tubulin and TUBB3.

### Quantitative image analysis

For the analysis of neurite length, initially at least 10 randomly taken images containing at least 100 cells were quantified using ImageJ and the NeuronJ plugin^70^. This semi-automated analysis was later replaced by image acquisition with the InCell Analyzer 2200 (GE Healthcare) and subsequent image analysis with the InCell Developer toolbox (≥100 cells per biological replicate obtained from 10–20 images). Growth cone analysis was conducted using a semi-automated CellProfiler script (≥100 cells per biological replicate obtained from 10–20 images) or an automated InCell Developer script (≥100 cells per biological replicate obtained from 10–20 images). Neurite outgrowth and growth cone data shown in Figs. 3 and 4 were generated from the same set of images. For the analysis of the growth cone areas after drug treatment, the growth cone area was normalized to the mean area of the untreated controls (Fig. 4C). Quantification of axonal swellings (>1 µm) was performed manually and normalized to the length of TAU1 positive axons as determined via ImageJ with the Neurite tracer macro^71^ (Fig. 2) or the InCell Developer toolbox (Figs 3 and 4). At least 100 cells, recruited from ≥10 randomly taken images were analysed per biological replicate.

For large scale image acquisition, cells were seeded into 96-well plates (µ-plates, ibidi; data shown in Figs 1, 3 and 4). For compound assessment (Fig. 4) positive and negative controls were included on each plate. Using the InCell developer toolbox, DAPI positive nuclei were segmented by object recognition. To separate nuclei within close proximity, an eroded image of the recognized nuclei was used as nuclear seed. Dead cells were excluded via size exclusion of nuclei. Neurites were identified by intensity segmentation of the TUBB3 channel. To avoid exclusion of fine neurites, the TUBB3 signal was enhanced prior to analysis. To exclude the soma and to count the length of single neurites and not the total neurite length of the cell, the nuclei signal was dilated (7 × 7 Kernel) and subtracted from the neurite channel. Neurite length was determined with an algorithm included in the InCell Developer toolbox. For growth cone analysis, object recognition was used on the actin channel. Only the area of signals co-localising with the neurite TUBB3 signal were included in the analysis. For the quantification of layer markers, DAPI, TBR1 and CTIP2 were segmented via object recognition and separated with a nuclear seed. Only TBR1 and CTIP2 signals co-localising with DAPI nuclei were counted. TBR1 and CTIP2 double positive cells were determined by co-localization of both markers with DAPI.

## Supplementary information

Supplementary information: Multiparametric rapid screening of neuronal process pathology for drug target identification in HSP patient-specific neurons

## Data Availability

The authors declare that the data supporting the findings of this study are available within the article and its supplementary information files. Parts of this study have been reported in the PhD thesis ‘Rapid semi-automated phenotypic assays for compound testing in patient-derived SPG4 neurons’ by Kristina Rehbach^72^.
