# Supplementary material for: Multiparametric rapid screening of neuronal process pathology for drug target identification in HSP patient-specific neurons
Source: Sci Rep. 2019 Jul 3;9:9615. doi: 10.1038/s41598-019-45246-4 (PMC6610147; doi:10.1038/s41598-019-45246-4)
Supplement: Supplementary file 1 — Supplementary information: Multiparametric rapid screening of neuronal process pathology for drug target identification in HSP patient-specific neurons [file 41598_2019_45246_MOESM1_ESM.pdf]

Supplementary information

Multiparametric rapid screening of neuronal process pathology for drug target identification in HSP patient-specific neurons

Kristina Rehbach[1,2,7], Jaideep Kesavan[1], Stefan Hauser[3], Swetlana Ritzenhofen[1], Johannes Jungverdorben[1,4,5], Rebecca Schüle[3,6], Ludger Schöls[3,6], Michael Peitz[1,4], Oliver Brüstle[1] *

[1] Institute of Reconstructive Neurobiology, University of Bonn Medical Faculty, 53127 Bonn, Germany

[2] Life and Brain GmbH, Life & Brain Center, 53127 Bonn, Germany

[3] German Center for Neurodegenerative Diseases (DZNE), 72076 Tübingen, Germany

[4] German Center for Neurodegenerative Diseases (DZNE), 53175 Bonn, Germany

[5] Memorial Sloan Kettering Cancer Center, 10065 New York, United States

[6] Department of Neurodegenerative Diseases, University of Tübingen, 72076 Tübingen, Germany

[7] Current address: Icahn School of Medicine, Mount Sinai, 10029 New York, United States

***** Correspondence to Oliver Brüstle: brustle@uni-bonn.de

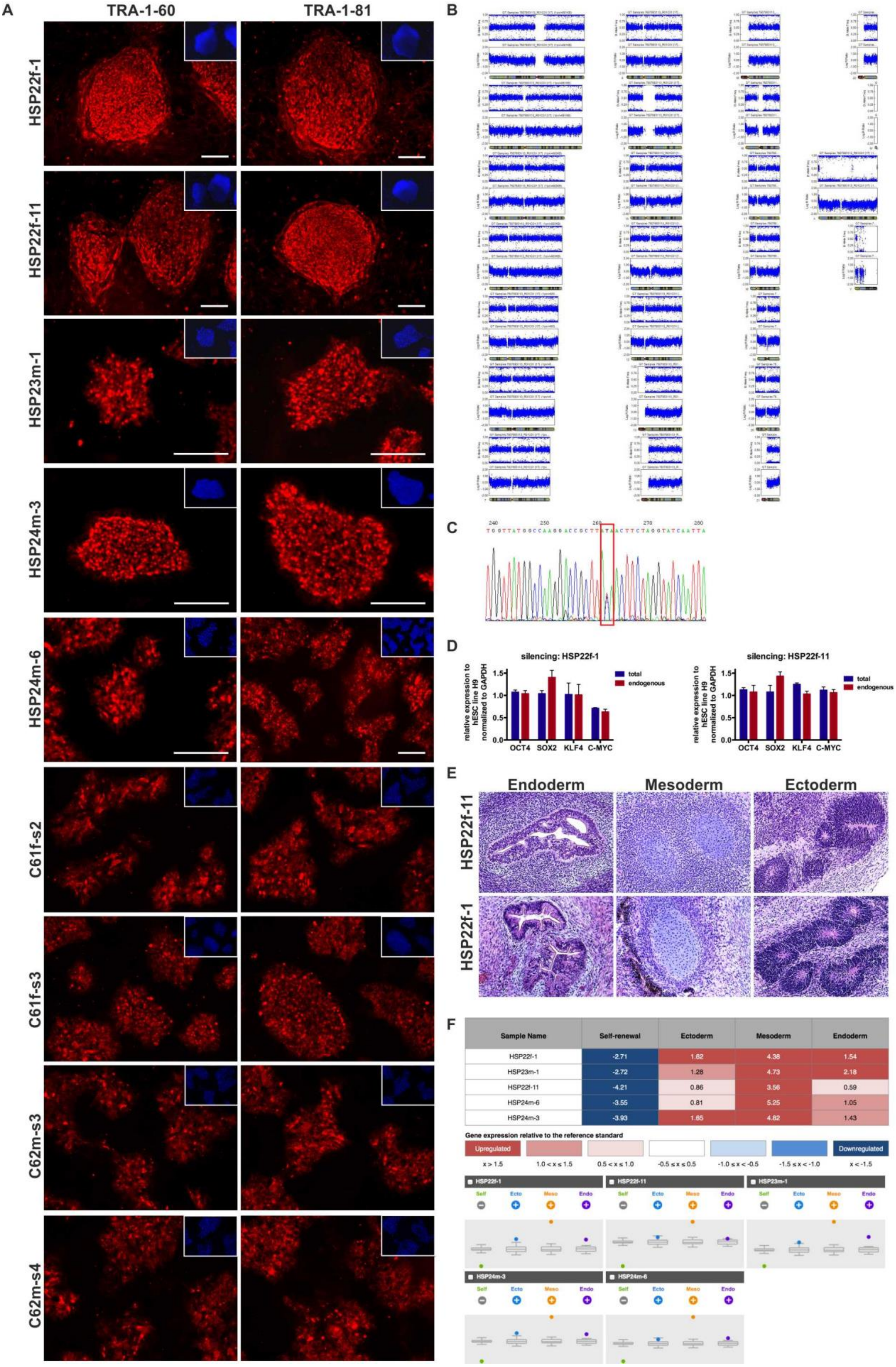
A
TRA-1-60
TRA-1-81
HSP22f-1
HSP22f-11
HSP23m-1
HSP24m-3
HSP24m-6
C61f-s2
C61f-s3
C62m-s3
C62m-s4
B
C
D
silencing: HSP22f-1
silencing: HSP22f-11
relative expression to hESC line H9 normalized to GAPDH
total
endogenous
OCT4 SOX2 KLF4 C-MYC
E
Endoderm
Mesoderm
Ectoderm
F
Sample Name | Self-renewal | Ectoderm | Mesoderm | Endoderm
HSP22f-1 | -2.71 | 1.62 | 4.38 | 1.54
HSP23m-1 | -2.72 | 1.28 | 4.73 | 2.18
HSP22f-11 | -4.21 | 0.86 | 3.56 | 0.59
HSP24m-6 | -3.55 | 0.81 | 5.25 | 1.05
HSP24m-3 | -3.93 | 1.65 | 4.82 | 1.43
Gene expression relative to the reference standard
Upregulated
Downregulated
x > 1.5
1.0 < x ≤ 1.5
0.5 < x ≤ 1.0
-0.5 ≤ x ≤ 0.5
-1.0 ≤ x < -0.5
-1.5 ≤ x < -1.0
x < -1.5
Self
Ecto
Meso
Endo

Supplementary Figure 1 IPSC validation

Assessment of pluripotency, genetic integrity, disease-causing mutation, silencing and differentiation potential. (A) All used iPSC clones express the pluripotency markers TRA-1-60 and TRA-1-81. (B) Exemplary image of SNP genotyping results used to ensure genomic integrity. (C) Confirmation of the donor disease association using Sanger sequencing. (D) Q-PCR confirms silencing of integrated retroviruses. (E) Subjecting the iPSC to a teratoma assay resulted in the formation of endoderm, mesoderm and ectoderm *in vivo*. (F) The teratoma *in vivo* assay was then replaced by directed *in vitro* differentiation into all three germ layers, followed by a TaqMan-based Score card assay. This Score card array also contains Sendai probes to confirm absence of Sendai virus, which would be depicted by a red flag if present.

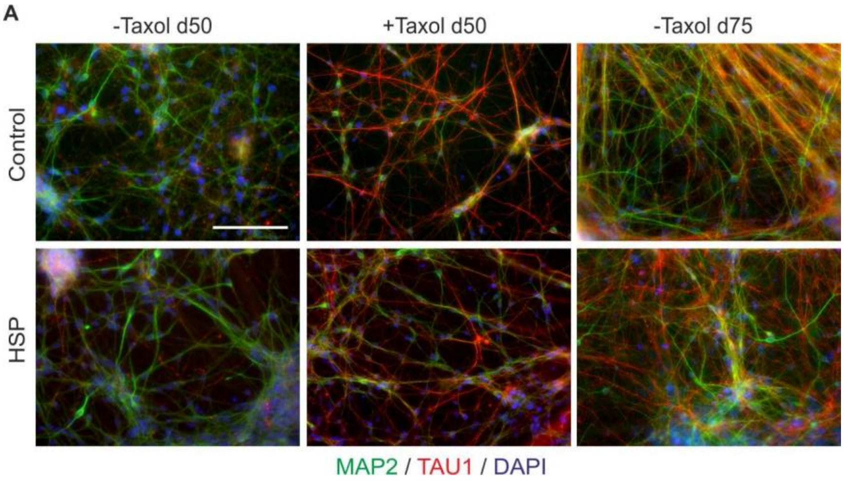

Supplementary Figure 2 TAU1 expression in cortical cultures

Taxol promotes the development of Tau1-positive axonal processes (A) Immunofluorescence staining against TAU1 (red) in day 50 cortical neurons demonstrates a lack of TAU1 positive structures, only dendritic MAP2 positive (green) structures are present. By day 75, TAU1 positive axons have been formed by control and SPG4 for neurons and built a very dense neuronal network. To be able to analyze cultures with lower density at an earlier time point, cortical progenitors were dissociated on day 44, followed by overnight treatment with 3nM taxol and subsequent culture until day 50. Scale bar: 100µm.

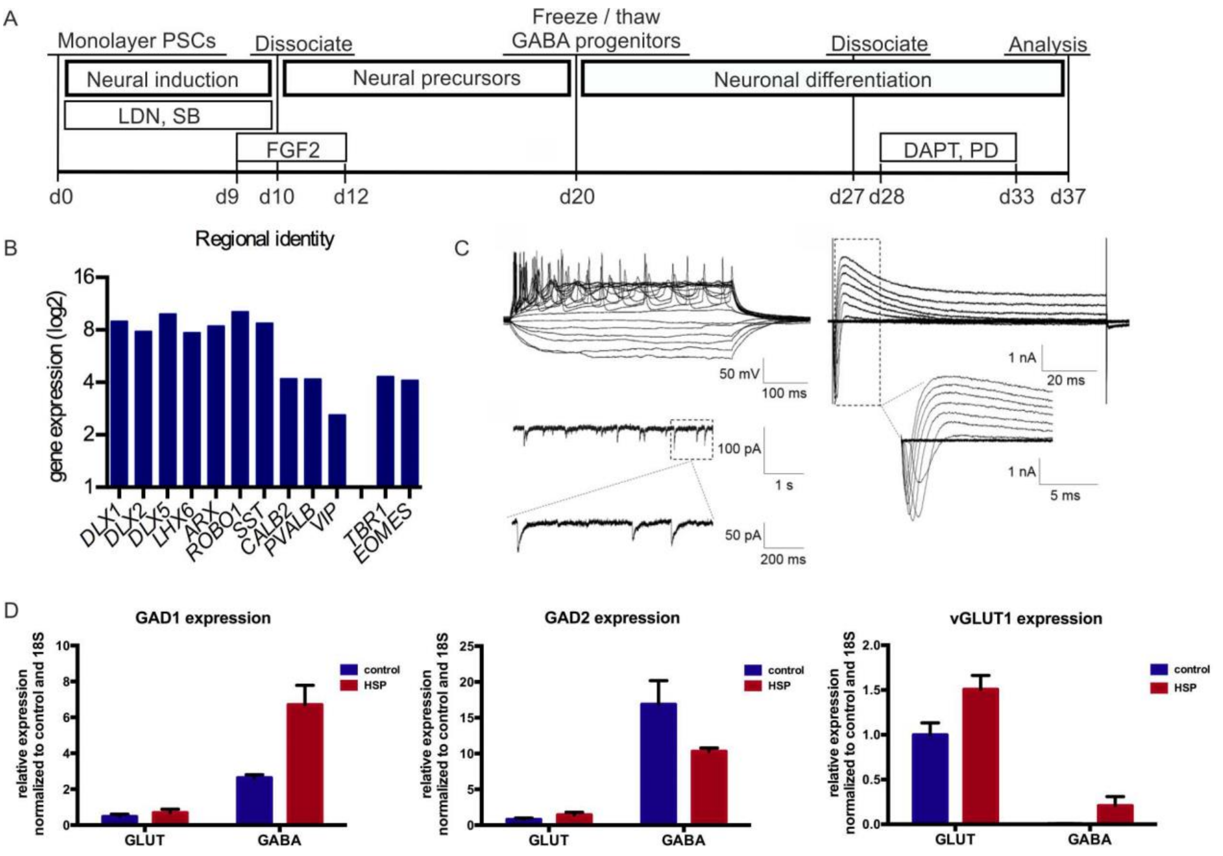

Supplementary Figure 3 Extended validation of GABAergic neurons

(A) Timeline of GABAergic differentiation. (B) Microarray (Affymetrix) expression data (log2) of two control GABAergic differentiation runs on day 37. Of the GABA subtype markers, only Somatostatin (*SST*) shows clear above-threshold expression at this time point. The GABAergic cultures further express the striatal markers *DLX1*, *DLX2*, *DLX5* and *LHX6* and the migration marker *ARX* and *ROBO1*. The forebrain markers *TBR1* and *EOMES* are not expressed above threshold levels. (C) Electrophysiological properties of GABAergic neurons. On the left, repetitive traces of action potentials recorded from cortical neurons at three months in vitro in response to 500 ms depolarizing current injection are shown (n=3). On the right, representative traces of fast inactivating inward current and sustained outward current in response to depolarizing voltage steps are depicted. Inset shows magnified view of the boxed area (n=3). In the lower left part, representative trace of spontaneous postsynaptic currents are presented. Expanded view of the boxed region is shown below (n=3). (D) Q-PCR of cortical glutamatergic cultures and GABAergic cultures. Control and SPG4 GABAergic cultures display higher levels of the transcripts of the GABA synthesizing enzymes *GAD1* and *GAD2*, whereas control and SPG4 glutamatergic cultures exhibit high levels of *vGLUT1* expression.

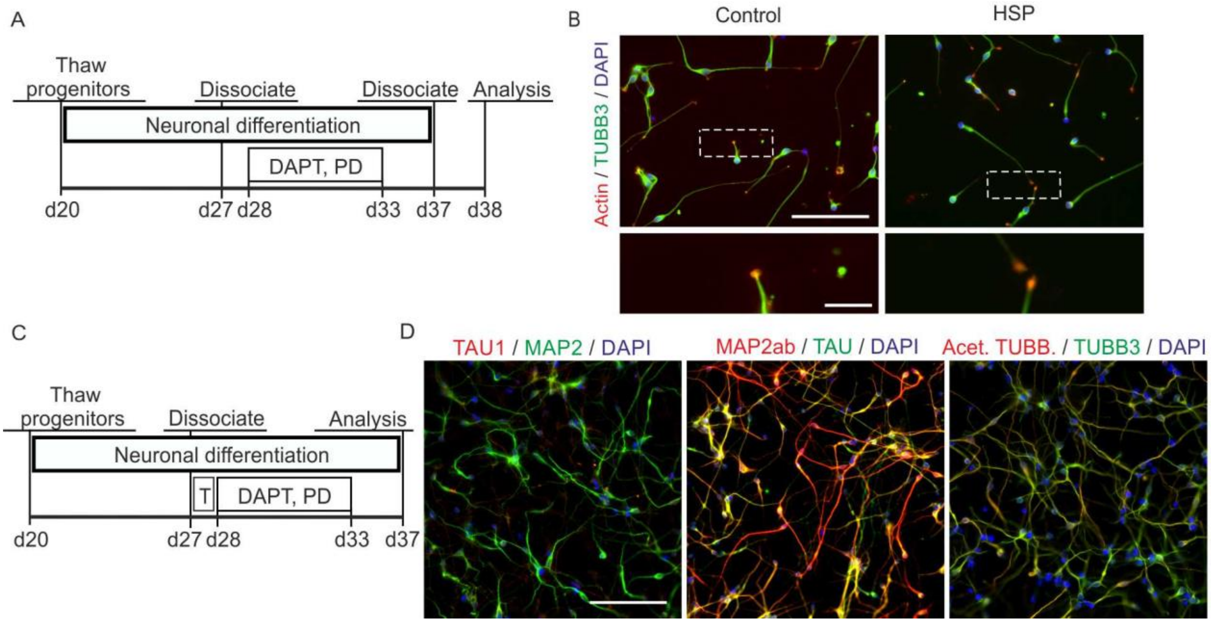

Supplementary Figure 4 Phenotyping of GABAergic neurons

(A) Timeline of neurite outgrowth and growth cone area assays with GABAergic neurons. (B) Immunocytochemistry against TUBB3 and actin on day 38. Scale bar: 50µm. Insets show magnifications of typical growth cones. Scale bar: 10µm. (C) Timeline of the axonal swelling assay in GABAergic neurons. T= 3nM taxol overnight. (D) Immunostaining against the axonal marker TAU1, total TAU and stabilized acetylated tubulin in TUBB3 positive neurons.

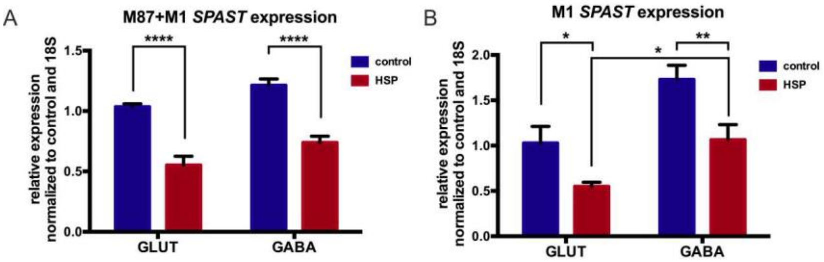

Supplementary Figure 5 Differential expression of M1 spastin in glutamatergic and GABAergic neurons

Q-PCR of cortical glutamatergic cultures and GABAergic cultures. (A, B) On q-PCR level, M87 and M1 *SPAST* expression is significantly reduced in SPG4 neurons compared to control neurons. (B) M1 *SPAST* expression is significantly higher in GABAergic patient neurons compared to glutamatergic neurons (control: n=6, HSP: n=8). 2way ANOVA, *: $p < 0.05$, **:$p < 0.01$, ***: $p < 0.0001$.

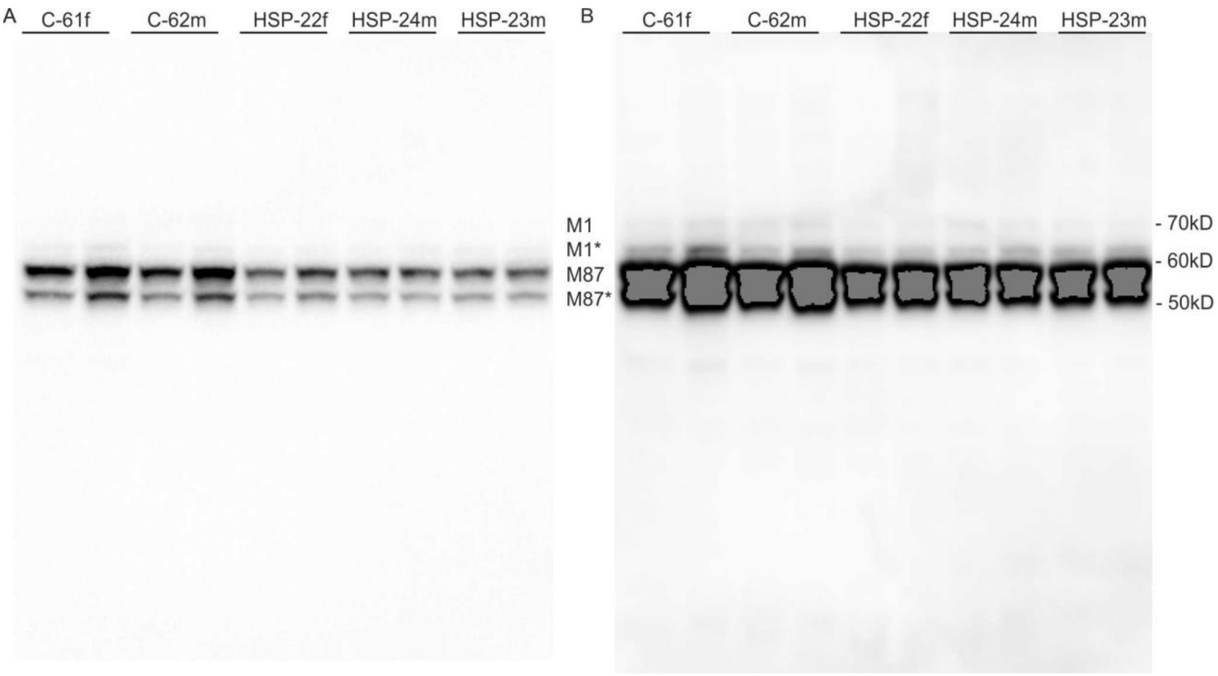

Supplementary Figure 6 Spastin Western Blots

Exemplary full spastin Western blot (A) after a short exposure time, showing only spastin isoforms M87 and M87* and (B) after prolonged exposure to show isoforms M1 and M1*.

Donor-ID	Age	Gender	Disease mutation	Age of onset	Virus
iLB-HSP-22f	40	female	UAG in exon 3 of *SPAST* gene c.557C>T	18	Retro
iLB-HSP-23m	44	male	UAG in exon 3 of *SPAST* gene c.557C>T	32	Sendai
iLB-HSP-24m	69	male	UAG in exon 3 of *SPAST* gene c.557C>T	40	Sendai
iLB-C-61f	49	female	wt	-	Sendai
iLB-C-62m	50	male	wt	-	Sendai
iLB-C-31f	23	female	wt	-	Retro

Supplementary table 1 Overview of iPSC lines

The overview table shows an overview of the iPSC lines used in this study, including the internal Donor-ID, the donor age of the time-point of fibroblast donation, the gender, details about the *SPAST* mutation, the age of onset of the disease and the type of virus used for reprogramming.

Clone name	Pluripotency markers	Absence of reprogramming factors	Genomic integrity	Pluripotent potential	Donor disease association
HSP-22f-r1	√	√	√	√	√
HSP-22f-r11	√	√	√	√	√
HSP-23m-s1	√	√	√	√	√
HSP-24m-s3	√	√	√	√	√
HSP-24m-s6	√	√	√	√	√
C-61f-s2	√	√	√	√	
C-61f-s3	√	√	√	√	
C-62m-s3	√	√	√	√	
C-62m-s4	√	√	√	√	
C-31f-r1	√	√	√	√	
C-31f-r3	√	√	√	√	

Supplementary Table 2 Overview of iPSC line validation

All used iPSC lines were validated according to pluripotency marker expression, the absence of reprogramming factors, genomic integrity via SNP arrays, pluripotent differentiation potential and sequencing of the disease-associated genomic region.

Tested small molecule	Concentrations	Neurite outgrowth	Growth cone area	Axonal swellings
Vinblastine	10nM	0.9953	0.3379	<0.0001
Latrunculin B	1nM	0.0080	0.0991	n.d.
	3nM	0.0010	0.0122	n.d.
	10nM	0.0017	0.0877	n.d.
DMH1	1µM	0.0964	0.0013	<0.0001
Dorsomorphin	1µM	0.0933	0.0016	<0.0001
GW3965	5µM	0.0038	0.0005	<0.0001
	10µM	0.9997	n.d.	n.d.
TRO19622	3µM	0.9989	0.0440	<0.0001
Scriptaid	1µM	0.9209	<0.0001	0.0005

Supplementary Table 3 Overview of compound assessment of all three short-term read-outs in cortical cultures

The table supplies an overview of the small molecules and concentrations used in the semi-automated compound assessment. Depicted are the p-values of the difference between untreated SPG4-conditions and SPG4 samples treated with tested small molecules. P-values shaded in green show a significant difference to the untreated SPG4 conditions, whereas fields shaded in red mark conditions, which had significantly altered values in control neurons. n.d.= not determined.

Supplementary Material

Immunocytochemistry

Cell cultures were fixed with 4% PFA for 10-15 minutes. For GABA fixation, the PFA solution was supplemented with 0.04% glutaraldehyde. Before staining, cultures were blocked with 10% FBS in PBS + 0.1% Triton X-100 for one hour. Primary antibodies were diluted in 10% FBS in PBS + 0.1% Triton X-100 and incubated over night at 4°C. For extracellular epitopes, the addition of Triton-X was omitted. Subsequently, fitting secondary antibodies were diluted in 10% FBS in PBS + 0.1% Triton X-100 and incubated for one hour. The following antibodies were used: Acetylated tubulin (ms IgG, Sigma, T7451, 1:1000; RRID:AB_609894), BRN2 (gt IgG, Santa Cruz, sc-6029, 1:500; RRID:AB_2167385), CTIP2 (rat IgG, abcam, ab18465, 1:500; RRID:AB_2064130), GABA (ms IgG, Sigma, A0310, 1:1000; RRID:AB_476667), MAP2 (rb IgG, Millipore, AB5622, 1:500; RRID:AB_91939), MAP2ab (ms IgG, Sigma, M1406, 1:500; RRID: AB_477171), TAU1 (ms IgG, Millipore, MAB3420, 1:500; RRID:AB_94855), TAU (rb IgG, Millipore, MAB10417, 1:300; RRID:AB_1977525), TBR1 (rb IgG, proteintech, 20932-1-AP, 1:1000; RRID:AB_10695502), TRA1-60 (ms IgM, Millipore, MAB4360, 1:500; RRID:AB_2119183), TRA1-81 (ms IgG, Millipore, MAB4381, 1:500; RRID:AB_177638), TUJ1 (TUBB3, ms IgG, Covance, MMS-435P, 1:1000; RRID:AB_2313773), TUJ1 (TUBB3, rb IgG, Covance, PRB-435P, 1:2000; RRID:AB_291637), vGlut1 (rb IgG, synaptic systems, 135303, 1:1000; RRID:AB_887875).

Quantitative RT-PCR

RNA was isolated using the Qiagen RNeasy Mini Kit. 2µg RNA were transcribed into cDNA via the iScript reverse transcriptase kit (Bio-Rad). QPCR was performed with the Taq DNA polymerase kit (Thermo Fisher Scientific), 18S was included as endogenous control.

**Primer target**	**Sequence**
18S forward	TTCCTTGGACCGGCGCAAG
18S reverse	GCCGCATCGCCGGTCGG
c-MYC endogenous forward	TTCGGGTAGTGGAAAACCAC
c-MYC endogenous reverse	CCTCCTCGTCGCAGTAGAAA
c-MYC total forward	AAGACTCCAGCGCCTTCTCT
c-MYC total reverse	TCTTGTTCCTCCTCAGAGTCG
GAD1 forward	CTTGTGAGTGCCTTCAAGGAG
GAD1 reverse	TGCTCCTCACCGTTCTTAGC
GAD2 forward	CTCGAAGGTGGCTCCAGTG
GAD2 reverse	CTCCCAAGGGTTGGTAGCTG

KLF4 endogenous forward	GACCAGGCACTACCGTAAACA
KLF4 endogenous reverse	CTGGCAGTGTGGGTCATATC
KLF4 total forward	CCCAATTACCCATCCTTCCT
KLF4 total reverse	ACGATCGTCTTCCCCTCTTT
M1 spastin forward	ACCCGCTGTTTGTAGGCTTC
M1 spastin reverse	TCTCATCCTCATCGATGCGC
M87+M1 spastin forward	GCGTCCGAGTCTTCCACAAA
M87+M1 spastin reverse	CCATTCCACAGCTTGCTCCT
OCT4 endogenous forward	GACAGGGGGAGGGGAGGAGCTAG
OCT4 endogenous reverse	GTTCCCTCCAACCAGTTGCCCCAAAC
OCT4 total forward	GTGGAGGAAGCTGACAACAA
OCT4 total reverse	TTCTCCAGGTTGCCTCTCA
SOX2 endogenous forward	GTATCAGGAGTTGTCAAGGCAGAG
SOX2 endogenous reverse	TCCTAGTCTTAAAGAGGCAGCAAAC
SOX2 total forward	GCCGAGTGGAAACTTTTCTCG
SOX2 total reverse	GCAGCGTGTACTTATCCTTCTT
SPAST Seq forward	CCACAACACCTGGCCTAAAG
SPAST Seq reverse	ACAGAGCAAGCGTCCATCTC
vGLUT1 forward	GGGCCATGACTAAGCACAAG
vGLUT1 reverse	CTCCTCGCTCATCTCCTCAG

Supplementary Table 4 Primer Sequences

Immunoblotting

For spastin protein immunoblotting, protein lysates were produced using RIPA Buffer supplemented with complete mini protease inhibitor cocktail (Roche). For SDS-PAGE, precast NUPAGE Novex 4-12% Bis-Tris Mini Gels were used with MOPS running buffer (Thermo scientific). 40µg protein were loaded per lane, and immunoblotted using anti-spastin antibody (Santa Cruz, sc-81624, 1:1000). As loading control, blots were stained with Ponceau solution (Sigma-Aldrich).
